# An analysis of the market for diving certificates

**DOI:** 10.3389/fspor.2025.1696686

**Published:** 2025-12-10

**Authors:** Frank Daumann, Julian Alexander Klöcker, Malte Schurade

**Affiliations:** Chair of Sports Economics and Health Economics, Friedrich Schiller University Jena, Jena, Germany

**Keywords:** diving certificates, SCP-paradigm, market concentration, SCUBA diving, market analysis

## Abstract

**Purpose:**

The market for recreational diving certificates is characterized by a small number of global providers and standardized products. Understanding how market structure influences firm behavior and market outcomes is essential for evaluating competitiveness and potential policy interventions. This study examines the market for diving certificates through the lens of the Structure-Conduct-Performance (SCP) Paradigm. It seeks to understand the role of entry barriers and market concentration in shaping competition and performance in this niche market.

**Methods:**

The analysis examines structural conditions and resulting market outcomes by using secondary data, industry characteristics and theoretical SCP reasoning. Special attention is given to network effects, proprietary training systems and certification standardization as sources of entry barriers.

**Results and Conclusion:**

Findings indicate that competition is limited by substantial entry barriers, enabling the dominant provider, PADI, to maintain considerable market power. Despite this concentration, technical efficiency and training quality remain consistently high across providers. However, monopolistic pricing strategies lead to allocative and distributive inefficiencies. Because training quality is maintained and consumer preferences are diverse, direct regulatory intervention appears unnecessary.

## 1 Introduction and problem definition

Recreational scuba diving with breathing apparatus in a maritime environment has developed from a marginal sport to a recreational activity that is now in high demand (1, 2). While diving was mainly used for commercial (sponge and pearl diving) and military purposes until the middle of the 20th century due to the limited technical possibilities and the resulting costs, this changed with the invention of the regulator (Georges Commeinhes, Émile Gagnan, Jacques Cousteau 1942/43) and the improvement of compressed air cylinders and diving suits. In the 1950s, the first diving safaris were organized by tour operators. Improvements in equipment (diving computers etc.) and corresponding media exploitation (feature films such as Abyss, Intoxication of the Deep etc.) led to diving becoming a mass phenomenon from the 1980s onwards. As diving, unlike swimming or snorkeling, is characterized by the entire body remaining below the surface of the water, i.e., it is a sport in a hyperbaric environment, it can have considerable negative consequences for the diver's health. For example, health problems can occur in the form of decompression sickness (the diver ascends too quickly), lung overstretching (the diver holds their breath as they ascend), nitrogen narcosis, oxygen poisoning and lack of pressure equalization (barotrauma) (3). Psychological problems such as panic or claustrophobia are also conceivable (4). Inadequate equipment or poor operation of the same can also lead to significant health risks. In addition, dangers to divers can arise from marine life (e.g., sharks, stonefish, cone snails). Finally, currents and waves pose considerable risks. To minimize such risks, both the athletes and their equipment must meet special requirements.

The identified risks of scuba diving for the health of the diver and the environment (3, 4) as well as legal requirements have led to diving centers that offer a comprehensive range of diving services (diving training, rental and sale of diving equipment, etc.) requiring proof of appropriate skills. This can be provided by the diver in the form of a certificate. In addition to the Open Water Diver (proof of the ability to dive with a diving partner to a depth of 18 meters), these include the Advanced Open Water Diver (to a depth of 30 meters) and various special certificates for deep diving, wreck diving, nitrox diving (Enriched Air Diver) and rescue diving (Rescue Diver) (5, 6). In the commercial sector, the Divemaster (qualification to lead dives) and Instructor (qualification to teach diving courses) are also offered.

A limited number of large, international recognized diving organizations such as the Professional Association of Diving Instructors (PADI), Scuba Schools International (SSI) and the National Association of Underwater Instructors (NAUI) are responsible for these diving certificates (5). This shows that PADI, as the world's largest diving agency, clearly outstrips the other providers in terms of the number of issued certificates. Against the background of this obvious concentration on the supply side, the question arises from an economic point of view as to the extent of competition in this market and, based on this, the question of the need for state intervention. In order to answer these questions, this article is structured as follows: First, the theoretical foundations of diving are conveyed and then the main areas of research in diving to date are presented. In the third section, the analytical tools—the SCP paradigm—are presented. Then, based on the SCP paradigm, the structure of the market for diving certificates, the behavior on this market and its market results are analyzed, before the fifth section explores any need for regulatory action. The article concludes with a conclusion and outlook.

## 2 Theoretical backgrounds

To classify diving, the criteria of type of breathing, objective and environment are used in particular. Regarding the type of breathing, a distinction can be made between apnoea diving (breath-hold diving), in which the diving process is carried out without technical aids for breathing solely by holding one's breath (7), scuba diving and saturation diving. While in scuba diving the diver remains underwater with breathing apparatus for a period of around one hour, saturation diving is characterized by the fact that the diver spends longer periods—sometimes days—in a pressure chamber, even at greater depths (8).

Research has dealt with the topic of diving from different perspectives. A central part of the scientific literature deals with scuba diving as part of the tourist offer, demand and the development of the location. On the one hand, the focus here is on travel behavior and the motivation of divers (9). This is directly followed by studies on market segmentation and consumer behavior. The aim here is to describe the different target groups within divers in a differentiated manner and to record their motivation, expectations and loyalty behavior (10–13). On the other hand, research in this area addresses the management of diving destinations (14, 15). Research questions focus on the tourism development of diving areas, the satisfaction of diving travelers and questions of infrastructure and experience design (16). Overarching studies in this area emphasize the economic importance of diving tourism, especially for island states and coastal regions in the global South (17), as well as the challenges posed by overtourism and uncontrolled resource use (18). The topics of environmental and sustainability research are closely linked to the first perspective mentioned. Their focus is on the effects of diving on marine ecosystems (coral reefs, seagrass beds and other sensitive habitats). These studies are usually characterized by an interdisciplinary approach and combine biological and social science perspectives (19, 20). The focus is on aspects such as identity formation, community experience, spirituality and experiencing nature. Diving may not only be understood as a sport, but also as a symbolically charged activity with cultural significance or as a medium of self-awareness (21–24). Gender issues and the portrayal of diving in the media are also occasionally addressed (25, 26). From a medical and sports science perspective, the physiological and psychological effects of diving are examined in particular. The focus here is on physiological stress (e.g., due to pressure, gas absorption or temperature differences) as well as possible risks such as decompression sickness, barotrauma or nitrogen narcosis [e.g., (27)]. In addition, there is a growing number of studies that deal with the health-promoting aspects of diving (28, 29). In connection with technical and safety issues, both equipment issues and accident analyses and preventive measures for diving are being investigated (3, 30, 118).

## 3 The SCP paradigm as a framework for analysis

The structure-conduct-performance paradigm (SCP paradigm) is an analytical instrument of industrial economics and goes back to the work of Mason (31) and Bain (32, 33). This instrument can be used to analyze the constitution of markets on the one hand and the interactions between the structure of the market, the behavior of market players and the market result on the other.

The SCP paradigm assumes a causal relationship between market structure, behavior and results. The market structure influences market behavior and this in turn influences the market result (32, 34). According to this paradigm, the structure of a market is determined by the particular characteristics of supply and demand. In particular, the existence and extent of barriers to market entry and exit, the extent of product differentiation, market growth and the number and size of market participants are understood as characteristics of the market structure (35). Market behavior, e.g., the behavioral pattern of companies in adapting to the markets in which they sell or buy (33), includes in particular the business objectives of the companies, the use of parameters like product, price and service. It also includes, for example, the extent of research and development (RandD) and cooperation or collusion behavior (35–37). Market outcomes, e.g., the economic results resulting from the behavior of companies (33), are understood to include aspects such as company profits or their ROI or return on equity, allocative and productive efficiency and innovative capacity (38). In its original conception, the paradigm suggests, for example, that higher market concentration leads to higher prices and higher profits [e.g., (34)]. Admittedly, the SCP paradigm is subject to considerable criticism, which has prompted the formulation of alternative explanatory approaches (39). In particular, it is criticized that the causal relationship between structure, behaveior and outcome is interpreted as too deterministic and that these three spheres rather influence each other (40). Furthermore, the lack of micro-foundation of the model is criticized; the strategic behavior of individual companies is not taken into account (41).

In this article, we ignore the causality assumption on which the SCP paradigm was originally based and interpret the paradigm merely as an instrument suitable for describing a market.

## 4 The market for diving certificates from the perspective of the SCP paradigm

In the following section, the market for diving certificates is analyzed using the SCP paradigm. To this end, the structure of the market is first examined before the behavior of market participants is discussed in detail. Finally, the market results are analyzed.

### 4.1 Key market players

To describe the structure of the market for diving certificates, it is necessary to characterize the key market players. There are essentially four different groups of players: In addition to the consumers, these are the diving centers or diving schools, the diving instructors employed by them or working on a freelance basis, and the providers of diving certificates. The consumers are predominantly private individuals who want to consume diving experiences and, due to the general conditions, strive for diving training whose completion is documented by an internationally recognized certificate—e.g., also by other diving schools (42, 43). The number of scuba divers worldwide in 2024 is estimated at between 6 and 9 million; according to estimates, 1 million people become certified divers every year (44).

There is comparatively low price sensitivity within the consumer group when using diving destinations (45, 46). However, it can be assumed that many potential diving students compare the prices for obtaining diving certificates from different providers in advance, although monetary criteria are not the only deciding factor. Reputation, safety standards and the worldwide recognition of the certificates are also likely to play a decisive role in the selection decision (5, 47). In this context, there is often an asymmetry of information, as consumers are generally not in a position to fully assess the actual quality of the training or the competence of the local instructors before starting a course. Confidence in the certifier's brand and third-party recommendations (e.g., via online platforms or travel providers) are therefore becoming increasingly important. Against this backdrop, the awareness and global recognition of certain certification companies play a particularly important role, as this is likely to have a significant influence on consumer behavior. Consumers tend to prefer certificates from these providers as they expect greater flexibility and acceptance for future diving activities worldwide. The reputation of certificate providers makes it easier for consumers to find their way in a market that is comparatively opaque in terms of quality (6).

Dive centers, of which there are currently around 10,000 worldwide (48), are multifunctional service providers that act as training centers. In addition to the course offerings relevant to certification, they often provide additional services such as the rental or sale of diving equipment, guided dives or personal advice (49). These additional services contribute to customer loyalty and differentiation from the competition. According to Dimmock et al. (50), dive centers offer education, equipment and experience. In their role as diving schools, diving centers provide the infrastructure and personnel required to carry out diving training. Their tasks include both the organization and carrying out both theoretical and practical training units (51). They often have a contractual relationship with a specific certification organization whose standards and training guidelines they implement. Structurally, diving centers are usually small and medium-sized businesses, which are mostly regionally oriented and operate in tourist regions such as coastal areas or vacation destinations. Their economic viability often depends on the season, with most of the demand being generated during vacation periods. A key characteristic of many diving schools is their close organizational connection to a specific certification company (50, 52). This relationship is often regulated via franchise or partnership models in which the diving school works exclusively with a certification organization. This relationship implies not only compliance with standardized training guidelines, but also the use of the brand's own training materials, examination formats and marketing materials (5). In a highly fragmented local market environment, dive centers are in direct competition with each other, with the main differentiators being pricing, the scope of services offered and the perceived quality of training. Reputation, customer ratings and recommendations play a key role in positioning in the market, particularly in light of the high interchangeability of standardized course formats. Overall, diving centers therefore form the central physical and economic platform for the realization of diving training. Their success depends largely on their ability to reconcile the requirements of the certification organizations with the expectations of consumers and at the same time successfully assert themselves in local competition (53).

Diving instructors are the third major group of actors on the market. Approximately 128,000 certified and actively working diving instructors are estimated to be operating worldwide in 2024 (44). As certified individuals, they are responsible for the operational implementation of training, both in theoretical lessons and practical exercises in the water (54, 55). Their work is fundamental to the implementation of the training standards set by the respective certification organizations. These instructors are typically either freelance or employed by diving schools, whereby a high degree of flexibility and mobility can be observed, particularly in tourist regions. Their forms of employment range from seasonal employment and project-based collaboration to self-employment with their own course offerings (55). The global structure of the diving market enables many instructors to work all over the world, particularly in vacation destinations with a high level of diving activity.

The key structural feature of the instructor role is a certificate-based professionalization in order to be allowed to work as an instructor (55, 56); hence it is necessary to acquire a corresponding instructor license. This license needs to be renewed regularly and requires continuous training and further education to master current standards, safety guidelines and pedagogical methods. This not only ensures the quality of training but also guarantees the instructors’ liability towards customers and certification organizations.

For the purpose of permanent certification, instructors are forced to pay annual license fees to the respective certification organization (55). This gives them the right to train under the brand of the respective provider and issue official certificates. This financial commitment is supplemented by an organizational dependency: instructors are bound by the rules and training standards of their certification organization, which stipulates the course content, examination requirements, number of dives and safety guidelines, among other things. According to Andy et al. (56), overall, diving instructors can be characterized as highly professionalized service providers who operate in a regulated environment structured by certification organizations. They contribute significantly to the implementation of quality standards and the learning success of consumers, but at the same time are dependent on the organizational and economic framework conditions set by the certifiers and diving schools.

Certification organizations as the fourth group of players form the normative and structural basis of the market for diving certificates (57). They not only define the content and safety standards of training but also create the conditions for the international recognition of diving certificates through their brand presence and institutional regulations (58). As cross-market bodies, they act both as private regulators and as market players with considerable influence on supply and demand. In addition to the organizations mentioned above (PADI, SSI and NAUI), CMAS (Confédération Mondiale des Activités Subaquatiques) is one of the largest international certification companies (5). PADI is in 187 countries and was issuing more than 25 million certificates between its founding in 1967 and 2017 (52, 59). The organization issued around 1 million certificates each year (60, 119). For the period from 2020 to 2024, approximately two million certifications are reported to have been issued by PADI, representing a significant decline in the overall number of scuba diving certifications. This trend is consistent with findings from the Business of Diving Institute (61), which indicates a decrease in PADI's market share in North America from 55% in 2023 to 43% in 2024, accompanied by an overall contraction of the market volume (62). In Western Europe, a similar pattern is observed, with PADI's share declining from 30% in 2022 to 27% in 2024.

PADI's harshest competitor SSI appears to be slightly smaller than PADI in terms of the number of certificates issued but clearly issues several hundred thousand certificates per year.

As we have seen above, other diving certification providers include CMAS, NAUI, SDI/TDI (Scuba Diving International/Technical Diving International), BSAC (British Sub-Aqua Club) and IANTD (International Association of Nitrox and Technical Divers), although these organizations are not as important as PADI and SSI. The central function of all these organizations is the development and updating of standardized training guidelines and monitoring compliance with them. A central feature is their licensing of instructors and diving schools (5, 50). Through a system of license fees, trademark rights and training materials, certifiers create a closed set of rules that controls and safeguards the delivery of training. They not only offer training materials and digital platforms, but also carry out evaluations, audits and training programs. Providers (diving schools, instructors) are usually tied to the certification organization via contractual agreements or franchise models, which enables extensive control of training quality and brand identity. Certifiers also play an important role in market communication. Through extensive marketing campaigns, international partnerships and online platforms, they significantly influence the visibility and attractiveness of their certificates for consumers. The strong brand orientation on the demand side means that globally active organizations such as PADI in particular have a dominant market position (119). This dominance is reflected not least in the global infrastructure of recognized diving centers, the availability of materials in many languages and the high number of active members worldwide (51).

Finally, certification organizations also act as institutional gatekeepers that decide on quality assurance in the market. In this context, however, it is essential to consider the role of the World Recreational Scuba Training Council (WRSTC), which functions as an international standards organization responsible for establishing and maintaining minimum training requirements in recreational scuba diving education and certification (63). WRSTC was created to ensure consistency, quality, and safety across training programs offered by various certifying agencies (63). Although the WRSTC itself does not issue diving certifications, its standards are adopted by most of the world's leading recreational diving organizations, including PADI, SSI, and NAUI. The WRSTC operates through a network of regional councils, such as the RSTC (United States), RSTC Europe, RSTC Canada, and RSTC Japan. This federated structure allows regional adaptation of training standards while maintaining a unified global framework (64). Since the certification organizations are themselves members of the various regional councils of the Recreational Scuba Training Council (RSTC), and the WRSTC merely defines the minimum training standards, the certification organizations are indirectly able to set and enforce training standards. In other words, they perform a regulatory function that is comparable to the traditional roles of professional associations or accreditation bodies.

At the same time, they secure their economic influence in the global diving market through their organizational networking with diving schools, instructors and training platforms (51). To obtain the required certificate, the interested party must contact a certified diving center or a certified independent diving instructor. In the case of PADI, this requires the following: The center must be officially registered with PADI and listed as a PADI Dive Center or PADI Resort (52). For becoming a PADI's certified diving center, the defined PADI safety and quality standards must be met, equipment that meets PADI requirements must be available and PADI-certified instructors must be employed. These instructors must be registered and insured with PADI. In addition, PADI sets standards with regard to the conduct of the courses, for example in terms of safety, course structure and maximum group size, and requires that the official PADI teaching materials (e.g., manuals, videos, online platforms) are used (52). To ensure that these standards are met, PADI carries out regular inspections. In addition, PADI enables its professional members to obtain specialized insurance coverage tailored to the unique risks of the diving industry. This includes options for professional liability, dive accident, and equipment or property insurance. These policies are designed to protect PADI instructors and affiliated dive centers against claims or financial losses associated with diving-related incidents (53). The professional liability insurance typically covers instructors and dive operators in cases where students or clients allege negligence, errors, or omissions during training or guided dives. It is often administered through PADI-endorsed insurance providers, such as Divers Alert Network (DAN), which offer regionally adapted coverage packages. Similarly, dive accident insurance provides divers with protection against medical and logistical costs following a diving injury, including emergency evacuation, hyperbaric treatment, and medical repatriation. PADI partners with these insurance providers to ensure that divers and professionals maintain continuous coverage that complies with PADI's risk management standards.

#### 4.1.1 Legal framework

If an accident occurs during a dive center's services, it will cause legal consequences, e.g., in the event of a breach of traffic safety obligations or negligent behavior. This applies to situations in which equipment is given to unqualified persons. Diving centers try to limit their liability by means of liability disclaimers. In the common tourist diving destinations such as Egypt, Australia, the Philippines, Thailand, the Maldives, Italy or Mexico to cite few at random, different access regulations apply for diving. For instance, Egypt does not have a separate law for recreational divers, but diving activities are channeled through indirect regulations. The “Chamber of Diving and Watersports” plays a central role here, regulating the commercial diving industry in cooperation with the Egyptian Ministry of the Environment. Only certified divers are allowed to dive independently, while so-called “Discover Scuba Diving” programs are permitted for inexperienced people, provided they are carried out under the direct supervision of a licensed instructor (120).

Although Thailand does not have a specific diving law, too, diving is regulated by local regulations in conjunction with international training standards (65). Most diving schools follow the guidelines of the major diving agencies PADI and SSI. Independent diving is also only permitted with a valid diving certificate (66). Like Thailand, there is no explicit national law in the Philippines that regulates scuba diving in the narrower sense. Nevertheless, there is de facto regulation through a combination of local regulations, environmental requirements and international safety standards (67). Most Philippine diving centers also work according to PADI or SSI guidelines and generally require proof of a recognized diving certificate for independent dives. Of particular relevance in the Philippines are the numerous marine protected areas where diving is strictly regulated. In these areas, for example, scuba diving is only permitted if it is done through an officially licensed diving center. In addition, permission from local environmental authorities is often required. As part of beginner programs, diving is possible without prior training, but only under the direct supervision of a certified instructor (68).

In the Maldives, recreational scuba diving is subject to formal government regulation. For example, the Maldives Recreational Diving Regulation and the Maldives Tourism Act (Law No. 2/99) set out minimum certification requirements, dive-center registration, maximum depth limits (30 m for all recreational diving), prohibition of decompression dives, and recognition of training agencies aligned with the Recreational Scuba Training Council (RSTC) standards. Additionally, detailed operational requirements for dive centers (rosters, supervision, logbooks) are outlined.

Italy has no specific national law dedicated exclusively to recreational scuba diving activities. Rather, diving is regulated via general maritime safety legislation and local laws (69).

In Mexico, there is a regulatory standard for diving operators issued by the Secretariat of Tourism. This standard applies to “tourist-recreational diving and underwater activities” and defines minimum safety and equipment requirements for operators. Another regulation addresses the certification of nature-tourism guides including diving guides. These show that Mexico uses indirect regulation of recreational diving (via tourism and service operator standards) rather than a dedicated diver-certification law (70).

In contrast to the former countries, Australia has one of the strictest legal safety standards in the field of scuba diving. Diving is regulated by government regulations and the requirements of semi-governmental institutions such as the Australian Diver Accreditation Scheme (ADAS). Independent dives are only permitted for people with a recognized diving certificate. However, inexperienced divers can gain their first experience as part of a trial dive under the direct supervision of a diving instructor (71).

Overall, regulations in the various countries require divers to provide appropriate proof of the necessary skills for recreational diving. Although diving without this certificate is possible within narrow limits, it is further restricted by the specific liability regulations of the service providers involved, such as diving centers and instructors.

#### 4.1.2 Barriers to market entry

There are considerable barriers to entry in the market for diving certificates, which pose major economic and structural challenges for potential new providers. These include reputation and trust in existing diving agencies: certification in diving is based on trust between diving schools, customers and insurance companies. As we have mentioned above, organizations such as PADI have built up a high level of credibility regarding the quality of their certificates through many years of market presence (53, 58). New providers face the challenge of gaining this trust, especially in safety-critical areas. On the other hand, accreditation and standardization represent a barrier to market entry: Diving certificates must comply with international standards, in particular ISO 24801 for diving training. Without recognition by umbrella organizations such as the RSTC (Recreational Scuba Training Council), market acceptance is nearly impossible to achieve. These regulatory hurdles act as institutional barriers to market entry, which pose major challenges for smaller or new market participants in particular. Furthermore, the global umbrella organization RSTC was largely co-initiated by PADI (116); since PADI is the largest market actor, it can be assumed that its influence in the umbrella organization is also currently significant. In addition, established providers have a global network of diving schools, instructor training centers, e-learning platforms and logistics systems (6). In some cases, the tangible and intangible infrastructure is owned by the certification organization; in other cases, the certification organizations have developed this infrastructure through contractual obligations. For example, many diving centers are exclusively tied to certain certification organizations, such as the PADI 5-Star Dive Centers (52).

On the one hand, new market participants would have to build up this infrastructure from scratch, which is associated with high investments. On the other hand, the contractual commitment and reputation for existing contract partners such as dive centers result in considerable switching costs. Both of these factors require new providers to make substantial investments. In this respect, there are very high barriers to entry for potential competitors in this market in the form of the required capital.

The reputation of existing providers combined with the global recognition of existing certificates, the establishment of internationally recognized training standards, the training of instructors according to these standards and the contractual commitment of the diving centers generate considerable network effects on the part of consumers (50). In order to enter this market, new providers would have to offer consumers an additional benefit that can only be generated—if at all—with substantial investment. Therefore, the market for diving certificates is characterized by high barriers to entry, which are the result of a combination of reputation, regulatory requirements, infrastructure dependency and network effects.

#### 4.1.3 Homogeneity of the good of “diving certificate”

Perfectly homogeneous goods are characterized by the fact that they are completely interchangeable from the consumer's point of view. This regularly goes hand in hand with high price sensitivity and a lack of brand loyalty (41). There appears to be a high degree of homogeneity in the case of diving certificates. For example, the basic certification (often called Open Water Diver) typically includes similar training content: Theory training, pool training as well as open water dives (6). All major diving organizations are based on international standards, such as the ISO standards and the specifications of the Recreational Scuba Training Council (RSTC). This standardization leads to a certain harmonization of content. In addition, the well-known providers in this market—PADI, SSI, NAUI and CMAS—offer comparable level models for training (e.g., Open Water Diver, Advanced, Rescue Diver, etc.), which suggests a degree of homogeneity (5, 72). Although the levels of certiﬁcations are fairly uniform, there are to some extent, significant differences in didactic methods, teaching formats (e.g., e-learning vs. face-to-face teaching), training style offered, type of equipment suggested, global recognition, philosophy and the cost structure of the courses (6, 54). Mainstream training agencies typically offer shorter entry courses designed to maximize accessibility and ensure safe recreational diving for a broader audience. These programs usually follow a modular model, beginning with a basic certification (Open Water Diver, OWD) to which additional specialty courses can be added subsequently, often at an additional cost. For example, PADI is characterized by its strong brand presence and consistent training structure, while CMAS focuses more on competency-based and sport-oriented training (73, 117).

In addition, there are certification organizations that take completely different approaches. For instance, Global Underwater Explorer's (GUE) Rec1 course is generally considered a very rigorous and cost-intensive entry-level program. It emphasizes buoyancy control, trim, team protocols, and nitrox use from the outset, introducing participants to technical-level skills and establishing a solid foundational competence. The course is guided by the principle of “begin with the end in mind,” aiming to develop divers with long-term proficiency from the very start (74).

Regarding training equipment, GUE introductory courses recommend investment in modular systems that can be adapted as the diver progresses. For example, the use of a backplate and harness system—rather than a conventional recreational buoyancy compensator device (BCD)—enables integration with a single-tank wing initially and later allows for adaptation to a larger wing configuration for twinset diving.

Since instructors have a strong influence on the quality of training, the quality of the final product is largely determined by the training and competence of the instructors. Instructor training requirements differ considerably among recreational scuba agencies, reflecting differences in philosophy, technical emphasis, and teaching methodology. PADI's Open Water Scuba Instructor (OWSI) program requires Divemaster certification, a minimum of 60 logged dives, and completion of an Instructor Development Course (IDC) culminating in an Instructor Examination, emphasizing safe recreational diving and standardized teaching protocols (75, 76). SSI instructors undergo a modular Instructor Training Program (ITP), focusing on student-centered teaching and flexibility in specialty courses, with both online and in-person components (77). NAUI instructors complete the Instructor Training Course (ITC), which stresses teaching methodology, safety, and instructor judgment, allowing greater flexibility in curriculum design (78). In contrast, GUE's instructor program is highly demanding, requiring extensive dive experience and emphasizing technical proficiency, team diving, and adherence to standardized procedures from the outset (79). GUE instructors are trained to maintain uniform technical standards across courses, integrating skills such as buoyancy, trim, and nitrox use from entry level. PADI, SSI, and NAUI programs prioritize accessibility and safe recreational diving, using modular structures that allow divers to add specialties progressively. Equipment training also differs, with GUE advocating modular systems, whereas mainstream agencies primarily use conventional recreational buoyancy control devices. These differences reflect broader organizational philosophies: GUE focuses on technical competence and exploration, while PADI and SSI prioritize broad participation and safety.

Overall, the product is only partially homogeneous, as the various certification organizations place emphasis on different aspects of training. In addition, they attempt to tailor their courses to specific educational priorities, focusing on areas such as safety, technical skills, team protocols, or environmental awareness, depending on their organizational philosophy. Besides that the certificate providers try to differentiate the product by offering additional certificates such as Wreck Diving or Night Diving, which are not required in individual cases.

#### 4.1.4 Market concentration

There is a total of twelve international certificate providers on the market for diving certificates, of which PADI dominates the market with a market share of around 75%. The next largest provider, SSI, has a market share of only 15%. While NAIU has a 5% market share, the remaining nine providers have an aggregate market share of just under 5% (61) (Table 1).

**Table 1:** Market shares in 2024.

Supplier	Market Share
PADI	≈ 75%
SSI	≈ 15%
NAUI	≈ 5%
BSAC	≈ 2%
SBI/TDI	≈ 2%
CMAS	≈ 1%
Others	≈ 2%

Source: own calculations based on Business of Diving Institute (61).

Using the Herfindahl index, the market concentration can be quantified with

$$H=\frac{1}{N}+N\sigma^{2}$$ (1)

Equation 1 shows the decomposition according to Hay and Morris (80), with N as the number of market actors and $\sigma^{2}$ as the variance of the market shares of the N actors. For the diving certificate market in 2024, this results in a Herfindahl index of nearly 0.65. Since a Herfindahl index over 0.25 is interpreted as a signal of a highly concentrated market and a value over 0.6 as an indication of a monopolistic market structure (81), the market for diving certificates has a value at 0.65 and therefore appears to have a monopolistic market structure. Due to the strong dominance of PADI, we assume that this organization has a quasi-monopolistic position in many tourist diving regions. This market power allows PADI to determine prices, training content and formats largely independently. Schmalensee (121) points out that such conditions can lead to smaller providers facing considerable barriers to entry, especially through network effects, brand loyalty and acceptance by dive centers worldwide.

#### 4.1.5 Interim conclusion

The market for diving certificates is highly concentrated: a few agencies, led by PADI, dominate through standardized training, franchise models, and strong global branding. They face many small, regional dive centers and freelance instructors who can differentiate only marginally in price, service, or reputation. Because consumers cannot easily assess training quality and diving involves safety risks, they rely heavily on trusted, international brands (6). As a result, most dive centers are tied to established agencies—around 6,000 of 10,000 worldwide are affiliated with PADI (48). Regulatory and insurance requirements further reinforce this dependence, making independent certification practically impossible in many regions. Network effects, global recognition, and high entry barriers restrict competition and leave new providers with little chance to gain market presence.

### 4.2 Behavior of market participants

#### 4.2.1 Pricing policy

The providers of diving certificates pursue a multi-layered and strategically differentiated pricing policy. Certification agencies generally apply fixed, centrally determined fees for the core elements of the certification process, including instructor training, annual instructor membership, student registration/certification and official training documentation. For example, to become a certified diving instructor with an organization such as PADI, a one-time registration fee (around 830 USD) must be paid, annual membership costs (around 460 USD) must be paid, and the corresponding teaching materials must be purchased (82). There is a standard registration/certification fee for each student diver, which is usually between 70 and 220USD (83). The costs for the instructor training itself (pre-courses are required, so there is a minimum of around 1,300USD (84). In addition, certification providers use tiered pricing for a wide range of course offerings (e.g., Open Water Diver, Advanced Open Water Diver, Divemaster), with prices typically increasing with the complexity of the course, costing 1700–3000USD for professional or specialty certifications (84). This approach capitalizes on consumers’ willingness to pay as they progress, aligning prices with the value of expert training and providing product differentiation within the training ladder (85, 86). Additionally, providers like PADI leverage their reputation regarding safety, standardization, and transferability of qualifications to enforce premium pricing (119). They can assume that divers or diving students are often willing to pay more for globally recognized certifications (22, 86).

While the dive agency fees are standardized, local course fees are very flexible. Dive centers in lower-cost regions such as Southeast Asia or Central America may offer all-inclusive courses for 230 to 360USD, while centers in Western Europe or North America may charge 500 to 700USD or more, reflecting regional differences in operating costs, seasonality and consumer purchasing power (44); Green, 2023; (87). This pricing allows direct providers of diving certification to remain globally competitive while maintaining consistent safety and educational standards (85, 86, 88). The design of the fees acts as a barrier to entry for less capitalized or informal providers. In this way, the agencies maintain an elite network of affiliated dive centers and instructors. This ensures compliance with brand-specific quality standards and at the same time minimizes price competition within their own partner network. As the agencies give the licensees—i.e., the dive centers—considerable leeway to adjust end customer prices to regional economic conditions and tourism demand, this allows for flexible market penetration without losing central control.

Nevertheless, minimum price recommendations and brand-specific awards (e.g., the PADI 5-star classification) set effective price floors and promote the bundling of product services. This leads to price stability, strengthens brand value and makes it more difficult for smaller agencies to gain market share through aggressive pricing. The combination of centralized price regulation and decentralized price adjustment corresponds to the model of monopolistic competition, in which the focus is on product differentiation rather than ruinous price competition (85).

#### 4.2.2 Quality policy

The major agencies—particularly PADI and SSI—typically employ strict, multi-layered quality assurance procedures that prioritize safety, standardized training, and the promotion of responsible diving behavior (89). In addition to the close collaboration with the umbrella organization for quality improvement, another central pillar of the agencies’ quality policy is the consistent structuring and continuous updating of the training curricula (6). These curricula are intended not only to impart technical diving skills and safety protocols but also to convey cognitive, conative, and affective attitudes that support responsible and ecologically sensitive behavior underwater (5). Regarding the delivery of content, the agencies increasingly rely on modern teaching materials that include e-learning modules (6).

Furthermore, the quality policy includes systematic procedures for the certification of diving instructors, their continuous professional development, as well as the introduction of access restrictions, such as high costs for accreditation and regular reaccreditation of the instructors (89). These measures ensure that only individuals with defined competence and attitude profiles are allowed to teach. This reduces the risk of inadequate training quality and strengthens the safety standards of the agencies.

The agencies regularly review and update their curricula and safety protocols based on empirical findings and accident analyses (6, 58). Requirements such as fit-to-dive assessments, special consideration for vulnerable groups (e.g., divers with chronic illnesses), and the inclusion of modules on accident prevention, causal analysis of accidents, and emergency response reflect a holistic, evidence-based approach to continuous quality improvement (90, 91). In addition, the diving agencies are jointly responsible for the development of standards and ISO certification (92).

A central element for stabilizing and expanding one's market position is continuous quality improvement (93, 94). Through continuous development and updating of internationally standardized curricula, instructor qualifications, and safety-related protocols, a globally uniform and predictable training quality is ensured (95). Since this high-quality training leads to a lower incidence of diving-related accidents, consumer trust in the respective brand is strengthened (90). The quality systems thus act as reputation-building mechanisms: Diving centers prefer internationally recognized certifications that are accepted equally by divers and regulatory authorities (96).

Furthermore, the certification pathways are modular and structured in a building-block manner (e.g., Open Water, Advanced, Specialty, Professional), which binds both diving students and instructors to agency-specific training tracks in the long term. These standardized learning paths create high switching costs, thereby promoting vertical integration and ensuring long-term customer loyalty.

In summary, it is evident that the mechanisms by which diving agencies implement and monitor quality control in training are highly variable and merit detailed consideration. One important element is the membership of mainstream agencies in the WRSTC, respectively in the RSTC, which function as umbrella organizations, promoting alignment around minimum training and safety standards across its member agencies. This ensures a baseline level of quality for recreational diving instruction, although the enforcement mechanisms remain largely self-regulated by individual organizations.

In contrast, GUE provides a clear example of differentiation within the industry. While not a member of the RSTC, GUE maintains a stricter curriculum and more rigorous evaluation criteria that are applied consistently across all instructor and diver courses (74, 79). For instance, to maintain certification, a GUE instructor must undergo an annual assessment, including both a health evaluation and physical endurance testing. Such requirements are intended to ensure that instructors demonstrate ongoing competence, technical skill, and readiness to teach safely at all levels.

By comparison, most mainstream recreational training organizations require instructors merely to pay an annual membership or renewal fee to maintain their teaching status, without formal reassessment of their practical or theoretical competencies. This distinction highlights a fundamental difference in safety culture and quality assurance across the recreational diving industry. The GUE model emphasizes continuous evaluation and skill maintenance, reflecting a higher benchmark for both instructor performance and diver safety. These variations underscore that quality policies in diving education are not uniform, and that the rigor of instructor assessment can significantly influence the overall quality of the training product delivered to divers.

#### 4.2.3 Product innovation

Certification agencies have substantially developed their educational portfolio in recent years. The resulting innovations go beyond the classic basic training and aim for an integrative connection of safety orientation, environmental awareness, digital transformation, and customer-centered further education. A central area of innovation is the introduction of environmentally focused specialized courses that specifically promote ecocentric attitudes and responsible behavior underwater (97, 98). In parallel, a significant digitization of teaching and certification formats can be observed. The implementation of e-learning platforms, mobile applications, and digital certificate management aims to make training processes more flexible and is intended to provide particularly tech-savvy user groups with location- and time-independent access to theoretical learning content (98). Another focus of innovative behavior is the integration of health-related content and risk prevention measures into the training logic. Against the backdrop of increasing knowledge about dive-related health risks—especially in connection with chronic illnesses and age-related limitations—many agencies have incorporated preventive medical modules into their programs e.g., (99). This includes structured health screenings, risk-adaptive training measures, and specialized course formats for the detection and management of typical diving (90, 91). Agencies develop location-specific certification models and environmental protection-linked qualifications in response to local environmental requirements and increased expectations for sustainable tourism practices. These include, among other things, training in benthic mapping, reef monitoring, or active participation in restoration measures (100, 101). In addition, the agencies expand their offerings to include experiential and specialized training courses such as underwater photography, technical diving, or night diving.

Through product innovations, the large agencies are able to establish additional barriers to market entry and secure their own market position. The aforementioned expansion of the offerings to include a variety of new course formats and specialized courses (e.g., marine conservation, technical diving), digital learning modules, and location-specific training on ecological features not only satisfies a changed, increasingly environmentally conscious demand behavior (98) but also enables strategic alignment with regulatory requirements for marine protected areas, tourism development plans, and sustainability-oriented travel markets (85, 100, 101). The integration of ecological competence profiles not only strengthens the environmental profile of the agencies but also opens up new fields of cooperation with public and private actors. In the same way, particularly the expansion of e-learning platforms and hybrid teaching formats, broadens geographical reach, addresses the needs of a younger, mobile clientele, and opens up customer segments that were previously served exclusively by local providers (102). This reduces logistical constraints while ensuring quality consistency through standardized content.

The expansion of the portfolio with niche-oriented offerings (e.g., apnea diving, underwater photography, risk management) promotes lifelong learning and thus customer loyalty. In this way, customer value is increased over the entire lifecycle, and switching costs also rise (22, 96).

#### 4.2.4 Interim conclusion

Overall, the major certification providers—PADI, SSI, and NAUI—have succeeded in securing and expanding their market power through a combination of structured pricing strategies, rigorous quality assurance, and continuous innovation for a long time. These interlinked strategies created effective barriers to market entry, fostered consumer and regulatory confidence, and ensured operational resilience in a changing ecological and tourist environment. High-quality training, combined with adaptive product development and carefully calibrated pricing, not only contributed to diver safety and environmental responsibility, but also reinforced the structural dominance of leading agencies in the global certification market.

Smaller, specialized organizations such as Global Underwater Explorers (GUE) are able to attract a dedicated segment of the diving market by offering more rigorous curricula, stricter evaluation standards, and a differentiated educational philosophy. By catering to divers seeking higher technical proficiency and standardized procedures, these organizations can incrementally capture market share from mainstream providers. Consequently, while major agencies retain broad structural dominance, the presence of smaller, quality-focused organizations introduces competitive diversity and may slightly reduce the market share of traditional leaders, particularly among highly committed or technically oriented divers.

However, market power is somewhat reduced by the fact that dive bases regularly recognize comparable dive certificates from the larger agencies, as they are based on the same training standards (ISO 24801). Nevertheless, certification organizations try to prevent this in some cases. For example, in early 2024, PADI dive centers in Africa and the Middle East were prohibited from offering dive training with certifications from organizations other than PADI (103).

### 4.3 Market outcome

To capture the market outcome, aspects like profitability, competitive dynamics and market efficiency are analyzed.

#### 4.3.1 Profitability

The total annual turnover of the diving certification market is estimated to be between 300 million and 470 million USD (61, 104). The estimated total profit of the certification agencies is likely to be in the order of 60 million to 180 million USD. PADI, as the largest provider, is estimated to generate annual revenues of 180 million to 300 million USD, with estimated profits of 30 to 110 million USD. These figures point to a solid profit margin, estimated to be between 20% and 40% of turnover, and reflect the strong market power of the company (73). SSI's annual revenues are estimated to be around 60 to 120 million USD and NAUI's around 20 to 60 million USD (105, 106). The profit margin of these two certification organizations is likely to be significantly lower than that of PADI. CMAS and the smaller providers are estimated to have an annual global turnover of around 25 million USD.

Such high margins are typical for industries with initial investments in the development of curricula, brand building, and a distributed network of instructors lead to scalable activities with low marginal costs per issued certificate. Once the fixed costs of training content and instructor accreditation are covered, the additional costs of certifying each diver—mainly administrative and material costs—are low.

#### 4.3.2 Competitive dynamics

The market for diving certifications has high barriers to entry, which are the result of several structural factors. Besides the consumers’ preferences and the information asymmetry on this market, it is brand awareness. PADI is perceived as the dominant brand in the leisure diving sector by both end consumers and dive centers (53, 60).

Additionally, the network of diving instructors and dive centers is hardly imitable for potential competitors: To achieve relevant market penetration, a large, geographically widespread network of affiliated dive instructors and partner dive bases is required. This process is resource-intensive and therefore only feasible for very potent potential new entrants.

Furthermore, training standards and safety protocols play a significant role. High standards are required in terms of training content, certification paths and safety, as diving is associated with inherent risks.

Due to these high barriers to entry, the emergence of new competitors is rare and usually limited to small, regional providers. In this context, the diving certification agency RAID (Rebreather Association of International Divers, now Dive RAID International) is worth mentioning, which was founded in 2007 and has expanded its course offerings significantly since then to include recreational and technical diving as well as freediving, and which operates internationally with the support of digital learning platforms (107).

#### 4.3.3 Market efficiency

In terms of allocative inefficiency, significant inefficiencies can be identified due to the monopolistic structures. The resulting welfare loss is reflected in the fact that potential customers whose willingness to pay is below the monopoly price are excluded from the market, and therefore too few production factors are channeled into the training of divers. At the same time, however, there is no evidence of qualitative inefficiency: the certificates issued by PADI and the other large providers meet internationally recognized standards, and the quality of training is generally considered to be high (89).

Lack of competition typically leads to less innovation and efficiency pressure, which in turn reduces technical efficiency (41). This can also be confirmed at least in part for the market under investigation: The market and especially the market leader show a high degree of process optimization through the use of online exams, digital course platforms, and automated certificate management (108). Moreover, the existing economies of scale are utilized due to the international presence. However, this is often denied to smaller providers like RAID. In addition, the overall innovation dynamics on the market are limited. Although PADI, for example, has introduced digital modules in recent years, many of these steps—such as fully digital courses—have been taken with a significant delay compared to competitors (109). However, due to limited reach and network effects, they usually remain in their market niche. In terms of distributive efficiency, PADI, due to its dominant market position, is able to implement first-degree price discrimination in order to maximize the willingness to pay of individual customers (diving instructors, diving schools, course participants). As the market leader, PADI uses its pricing flexibility and is able to charge prices that are above the competitive level. This excludes certain groups of diving bases and end consumers from the market.

#### 4.3.4 Interim conclusion

The diving certification market is highly concentrated, with limited competition. PADI's quasi-monopolistic position enables stable, above-average profits and low marginal costs, reflecting a scalable business model grounded in brand strength, standardized training, and a global network. High entry barriers suppress competitive dynamics; innovators such as RAID introduce new formats but lack the reach to gain economic relevance.

Allocative inefficiencies arise because prices exceed marginal costs, restricting access and reducing overall welfare. Besides that, pricing strategies exploit willingness to pay and exclude less affluent customers. Technically, however, the market leader operates efficiently through digital processes and economies of scale (53, 119), while dynamic efficiency remains weak, as innovations originate from smaller niche providers and are adopted late.

In sum, the market is highly profitable for the dominant agency, but structurally uncompetitive and only modestly innovative. PADI's market power delivers short-term efficiency gains, yet limits long-term innovation and promotes an imbalanced market distribution.

## 5 Assessing the necessity of government intervention

The evaluation of the necessity for government intervention should consider the following two key aspects: On the one hand, major certification providers operate on a global scale, thereby limiting the potential impact of intervention at the national level. Conversely, the necessity and design of government intervention are contingent upon the specific competitive model employed. An approach that is based on Austrian Economics would certainly lead to the conclusion that there is no need for action at all, but rather that the market dominance of PADI is only temporary and should be leveled out after a certain period of time due to evolutionary processes (110–112). By contrast, an antitrust policy grounded in the model of perfect competition would employ instruments designed to eliminate dominant market positions—potentially even through the structural dissolution of the organization in extreme cases (113, 114).

A pragmatic approach would aim to reduce PADI's market power (115). A suitable package of measures should address the mechanisms that PADI uses to maintain its dominance—such as its high brand awareness, its widespread partnerships, the high switching costs, and the vertical integration of its training and certification system. A first step in this direction would be to standardize and promote the mutual recognition of certifications between different certification organizations. While mutual recognition of certifications between the larger certification organizations is already partially in place for some consumer training levels, there are still many obstacles in place for instructor training. By introducing additional optional standards—particularly in the area of instructor or dive instructor training—and by making it a requirement to mutually recognize training based on these standards, switching costs would be significantly reduced, thereby reducing the benefits that PADI gains from vertical integration and exclusive “pipeline” training models. A second step could be to address the educational resources and training materials. PADI's market position is strengthened by its control of the training materials, which closely link diving theory, practical exercises, and environmental protocols. This would allow independent diving instructors and smaller agencies to offer training that meets strict safety and environmental criteria without the burden of restrictive brand licensing. Open educational resources would expand the market capacity for innovation and differentiation (for example, local eco-modules or indigenous conservation practices), thereby reducing PADI's dominance as the sole source of supply.

Diversifying regulatory recognition and tourism partnerships could be a third approach. In many protected areas and dive destinations, PADI or companies affiliated with PADI tend to be given preferential access, operator status, or marketing partnerships. Such institutional recognition strengthens the tie to a particular organization and inhibits diversification. In many countries, such as Croatia, France or Italy, regulatory authorities formally recognize scuba diving certifications issued by agencies that comply with international ISO standards. This recognition extends beyond purely recreational purposes and often functions as a prerequisite or credential for employment in public or professional diving activities, such as scientific, environmental, or underwater archaeological work. The adoption of ISO-aligned certification frameworks provides facilitating mutual recognition of qualifications and intensifies competition between the certification organizations. Therefore, this approach should be extended to other countries which would significantly reduce the barriers to entry for smaller certification providers.

However, the market for diving certificates is very differentiated and the needs of the customers vary greatly (9, 13), which eases new certificate providers to enter the market by focusing on these target groups. Moreover, there is likely to be little willingness on the part of dive centers to turn away potential customers with “fake” certificates. This scenario is particularly probable in regions characterized by intense competition among dive bases, especially in locations that are both highly attractive and frequently visited. Finally, the diving scene appears to be highly interconnected, as evidenced by the substantial presence of specialized dive magazines and blogs. Consequently, issues related to service quality are likely to prompt swift customer responses and informal sanctions. All in all, there is a considerable scope for dive centers to react to excessive price demands by certificate providers. Therefore, from the perspective of a dynamic understanding of competition, there is no need for regulatory intervention.

## 6 Conclusion and outlook

The analysis of the global scuba diving certification market reveals a highly concentrated market structure, characterized by a quasi-monopolistic structure with a dominant player—PADI. This market structure generates an ambivalent dynamic: On the one hand, standardized training formats and internationally recognized safety protocols ensure a high level of training quality and consumer trust. On the other hand, the market-dominant position of PADI results in significant distortions of competition, which manifest themselves in price-setting leeway, delayed innovation, and limited market penetrability.

With an estimated market share of around 75% and a corresponding HHI at 0.65, PADI leverages its structural advantages—such as a dense global network of partner dive centers, exclusive training structures, and significant economies of scale—to secure its dominant position. Pricing is based on first-degree discrimination well above the marginal cost, which leads to allocative inefficiency. At the same time, the pressure to innovate is low, as new educational formats and digital offerings are primarily driven by smaller players such as RAID, whose market reach is limited by existing entry barriers. Despite the structural competitive disadvantages, the market overall ensures a high level of technical efficiency. Compliance with international standards and national diving laws in important destinations (e.g., Egypt or Australia) helps to minimize safety risks. In an environment characterized by market-side information asymmetries, brand reputation (especially that of PADI) serves as a crucial signal of trust and significantly influences the consumer's purchasing decisions.

While we see no market failure in the sense of complete inefficiency or insufficient supply, regulatory approaches can still be identified that aim to strengthen the competitive dynamics of this market without compromising the existing training quality.

## Data Availability

The original contributions presented in the study are included in the article/Supplementary Material, further inquiries can be directed to the corresponding author.
